# Design of a Multi-Mode Mechanical Finger Based on Linkage and Tendon Fusion Transmission

**DOI:** 10.3390/biomimetics8030316

**Published:** 2023-07-17

**Authors:** Yi Zhang, Qian Zhao, Hua Deng, Xiaolei Xu

**Affiliations:** 1 College of Mechanical & Electrical Engineering, Central South University, Changsha 410083, China; zhangyicsu@csu.edu.cn (Y.Z.); gxdx_zhaoqian@163.com (Q.Z.); xuxiaolei@csu.edu.cn (X.X.); 2 State Key Laboratory of Precision Manufacturing for Extreme Service Performance, Central South University, Changsha 410083, China

**Keywords:** mechanical finger, underactuated, adaptive, coupled, multi-mode

## Abstract

Today, most humanoid mechanical fingers use an underactuated mechanism driven by linkages or tendons, with only a single and fixed grasping trajectory. This paper proposes a new multi-mode humanoid finger mechanism based on linkage and tendon fusion transmission, which is embedded with an adjustable-length tendon mechanism to achieve three types of grasping mode. The structural parameters of the mechanism are optimized according to the kinematic and static models. Furthermore, a discussion was conducted on how to set the speed ratio of the linkage driving motor and the tendon driving motor to adjust the length and tension of the tendon, in order to achieve the switching of the shape-adaptive, coupled-adaptive, and variable coupling-adaptive grasping modes. Finally, the multi-mode functionality of the proposed finger mechanism was verified through multiple grasping experiments.

## 1. Introduction

Robotic hands are essential end effectors in robotic systems and have been widely used in the manufacturing, service, and rehabilitation industries. Humanoid robotic hands are usually developed to imitate the dexterity and versatility of human hands [1,2], which have high dexterity and can perform different grasping actions on target objects according to their shape, size, and relative position [3,4].

Some scholars have statistically classified the grasping action types of human hands, such as Feix et al. [5] and Liu et al. [6], who focused on the static and stable grasping of a single hand and, according to (1) opposition type, (2) the virtual finger assignments, (3) type in terms of power, precision, or intermediate grasp, and (4) the position of the thumb, divided the hand’s grasping actions into 33 types. Stival et al. [7], based on the previous classification methods, collected electromyography and kinematic data during human grasping actions to establish the kinematic classification and muscle classification methods for human hand actions. In summary, the grasping ability of the human hand is diverse to adapt to objects of various sizes and shapes. All of this has put higher demand on humanoid robotic hands’ dexterity and grasping mode diversity.

In recent years, to achieve a better imitation of human hands, scholars have developed numerous dexterous humanoid robotic hands [8]. For example, Cheng et al. [1] proposed an adaptive humanoid finger with a nine-bar mechanism; the I-limb robotic hand [9] by Touch Bionics; the Hannes hand [10] by M. Laffranchi et al.; the BeBionic Hand by Ottobock, etc. However, the above robotic hands all use coupled-adaptive underactuated mechanisms, in which two or three finger joints of a finger bend or extend simultaneously with a fixed coupling ratio under the drive of a single motor. Although the underactuation simplifies the mechanism and reduces the weight to a certain extent, it sacrifices the robotic hand’s dexterity and grasping mode diversity, making the robotic hand’s phalanges unable to move independently or in a coupled manner like the human hand. Moreover, the robotic hand’s interphalangeal coupling ratio is fixed and cannot be changed with the object’s characteristics, resulting in limited grasping modes of the robotic hand.

In terms of the interphalangeal coupling ratio, researchers have carried out a lot of exploration. The ILDA hand [11] by Kim et al. consists of a linkage mechanism, three motors, and three ball screw nuts for each finger, which can achieve the independent and combined grasping of the three finger joints of the robotic hand through the linear displacement of the ball screw nut pairs. However, the complex linkage mechanism and large quantity of parts are not conducive to installation and maintenance in the later stage; furthermore, the interphalangeal coupling ratio is fixed and unchanged. Quintero et al. [12] captured and analyzed a tennis ball spinning on top of a flat surface by the subject’s right hand using a Vicon optical motion capture system, and they found that the human hand’s interphalangeal coupling ratio was changing dynamically rather than being fixed during the process. Therefore, making the robotic hand’s phalanges able to move independently or changing the coupling ratio dynamically can improve robotic hands’ dexterity and grasping mode diversity.

In addition, the linkage transmission and tendon transmission mechanisms are currently the most widely used in robotic hands. For example, linkage transmission mechanisms have been adopted by the Harbin Institute of Technology [1], the German Aerospace Center DIL [13], the German Schunk Hand, and Ottobock’s BeBionic Hand [14]. However, the linkage transmission mechanism has weaknesses, such as complex structure and limited flexibility. Meanwhile, tendon transmission mechanisms have been adopted by the Huazhong University of Science and Technology’s X-hand [15], the UK’s Shadow Hand, the I-Limb Hand, and the American Washington Hand. However, the tendon transmission mechanism has a defect of insufficient rigidity, as its tendons are easy to break and require the application of pre-tightening force [8]. Some researchers [16] have tried to integrate the linkage and tendon mechanisms in recent years. Inspired by the above work, we attempted to design a new transmission mechanism that combines the advantages of solid rigidity, stability, and high transmission efficiency of linkage transmission with the benefits of the flexible, lightweight, and simple structure of tendon transmission.

This paper proposes a multi-mode humanoid mechanical finger based on linkage and tendon fusion transmission. The finger comprises two motors. The linkage mechanism is driven by the main motor as the finger’s power input; the tendon mechanism mainly acts as a coupling mechanism to make the finger achieve humanoid coupling motion. Moreover, the finger’s interphalangeal coupling ratio can be dynamically changed based on a minor motor to adjust the tendon’s length and tension. Therefore, the finger can realize shape-adaptive, coupled-adaptive, and variable coupling-adaptive grasping modes simultaneously.

The rest of this paper is organized as follows: Section 2 introduces the mechanical design of the finger. Section 3 analyzes the kinematics and statics of the finger. Section 4 explains the control strategy of the three modes. The performance of the finger is evaluated in Section 5, and the conclusions are discussed in Section 6.

## 2. Design of The Multi-Mode Mechanical Finger

This section first introduces the mechanical design of the finger’s linkage mechanism and tendon mechanism. Then, we present the design principle and motion characteristics of the finger’s shape-adaptive, coupled-adaptive, and variable coupling-adaptive grasping modes.

### 2.1. Overall Mechanical Design

**Figure 1:**
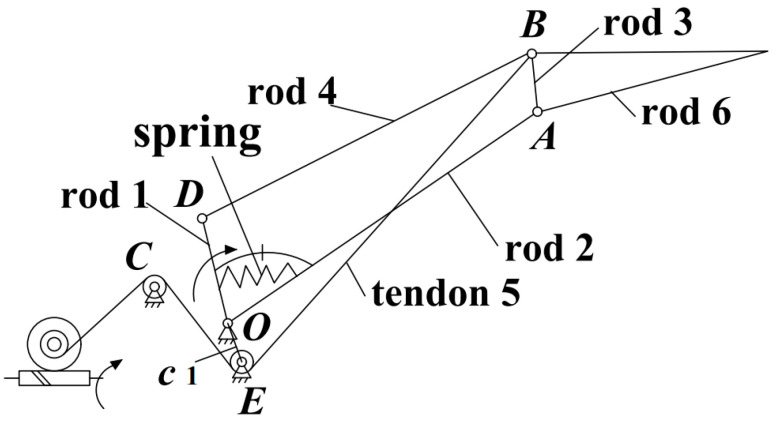
Multi-mode humanoid mechanical finger, which is composed of two parts: linkage mechanism (linkages 1-2-3-4-6, spring) and tendon mechanism (tendon 5, worm gear, and worm).

For a humanoid robotic hand, appropriate size and weight are as important as torque and speed performance. Reduced robotic hand size allows for better cosmesis for a broader population of users, while low weight is more comfortable when the device is worn for extended periods [17,18]. To achieve a more lightweight structure, we consider the middle phalanx and distal phalanx of the human finger as a unit and designed the humanoid finger, as shown in Figure 1. The finger is composed of two parts: the linkage mechanism and the tendon mechanism. Linkages 1-2-3-4 constitute the proximal phalanx’s main structure, and link 6 constitutes the distal phalanx. One end of tendon 5 is wound on the tendon wheel coaxial with the worm gear, and the other end is fixed at point B after changing the direction with shafts C and E. When tendon 5 is in a tension state, tendon *BE* (a section of tendon 5) is equivalent to a rigid linkage, and *c*1 is a fixed bracket, so *c*1-2-3-5 constitutes a coupled four-bar linkage mechanism [19]. The main motor drives rod 1, transferring power to the phalanxes. The power is first transmitted to the proximal phalanx’s rod 2 by the spring and mechanical limit and then transmitted to the distal phalanx’s rod 6 by rod 4. The tendon driving motor adjusts the tendon *BE’s* length and tension via a worm gear mechanism.

### 2.2. Finger’s Multi-Mode Design

The coupling ratio between the finger’s proximal phalanx and distal phalanx is mainly related to the tendon *BE’s* length in the linkage mechanism *c*1-2-3-5. In addition, there is no coupling relationship between the proximal phalanx and distal phalanx without constraint on tendon *BE*. The tendon driving motor adjusts the tendon *BE’s* length and tension via the worm gear mechanism, allowing the finger to switch between shape-adaptive, coupled-adaptive, and variable coupling-adaptive grasping modes.

#### 2.2.1. Shape-Adaptive Grasping Mode

**Figure 2:**
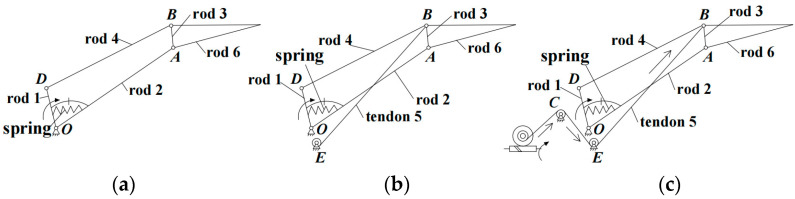
Finger multi-mode principle. (**a**) Shape-adaptive grasping mode: tendon ineffective, without coupling relationship. (**b**) Coupled-adaptive grasping mode: tendon keeps tensioning, the coupling ratio is fixed. (**c**) Variable coupling-adaptive grasping mode: the coupling ratio changes with the tendon’s extension.

When the tendon is in a relaxed state, tendon *BE* in the coupled linkage mechanism *c*1-2-3-5 will be ineffective, and only the 1-2-3-4 four-bar linkage continues to function. Therefore, the finger will transform into the shape-adaptive grasping mode [8], as shown in Figure 2a. In this mode, the proximal phalanx and distal phalanx will bend or extend simultaneously under the driving of the linkage driving motor, without relative motion before contacting objects.

#### 2.2.2. Coupled-Adaptive Grasping Mode

As shown in Figure 2b, the *c*1-2-3-5 and 1-2-3-4 mechanisms work in this mode. This mode needs tendon 5 to remain in a tension state; then, tendon *BE* will be equivalent to a rigid linkage. The proximal phalanx and distal phalanx will move with a fixed coupling ratio before contacting objects. When the proximal phalanx contacts objects and stops moving, the tendon driving motor starts to rotate, causing tendon 5 to extend and release a degree of freedom, allowing the distal phalanx to continue moving until enveloping the object.

#### 2.2.3. Variable Coupling-Adaptive Grasping Mode

Tendon *BE’s* length change will affect the coupling ratio between the proximal phalanx and distal phalanx. When the tendon driving motor rotates in a specified relationship with the linkage driving motor, tendon *BE’s* length will be stretched under the tension of point *B*, leading to a change in the coupling ratio. As shown in Figure 2c, the entire mechanism becomes a variable coupling-adaptive mechanism. The proximal phalanx and distal phalanx will move with a changing coupling ratio before contacting objects.

## 3. Finger Kinematics and Statics Analysis

### 3.1. Kinematics Analysis

To ensure the proposed finger mechanism can achieve humanoid operation, it is necessary to analyze the kinematics and mechanical characteristics of the finger. The structural parameters of the finger can also be determined via the analysis results [20]. The variable coupling-adaptive mode is achieved by adjusting tendon *BE’s* length based on the coupled-adaptive mode. Therefore, we take the coupled-adaptive mode as an example to analyze.

**Figure 3:**
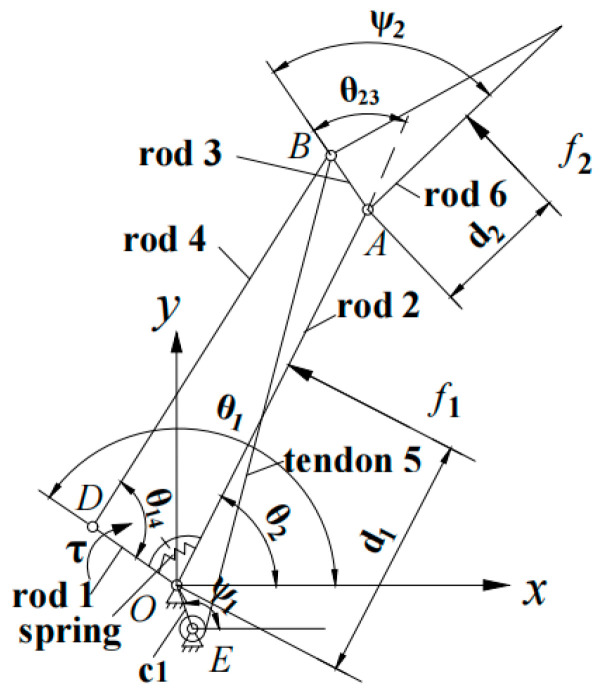
Coordinate system and structural parameters of finger mechanism.

As shown in Figure 3, the origin is set at point *O*, $l_{i}$ is the length of the *i*th link rod (*i* = 1, 2, …, 6), and $\psi_{i}$ is the constant angle determined by the mechanical structure, where $\psi_{1}$ is the angle between *c*1 and the positive half of the *x*-axis, and $\psi_{2}$ is the angle between $l_{3}$ and $l_{6}$. $\theta_{i}$ is the angle between the *i*th rod and the positive half of the *x*-axis, and $\theta_{ij}$ is the angle between $l_{i}$ and $l_{j}$. Establish the expression for the closure-equation link polygons OABE (including linkages *c*1-2-3-5) and OABD (linkages 1-2-3-4), and then rotate the coordinate system O-*XY* around point *O* by an amount $-\theta_{2}$, leading to [19]:

(1) $$l_{3}e^{i\theta_{23}}+l_{4}e^{i\theta_{24}}=l_{1}e^{i\theta_{1}}+l_{2}$$

(2) $$l_{3}e^{i\theta_{23}}+l_{5}e^{i\theta_{25}}=l_{c1}e^{i\left(\psi_{1}-\theta_{2}\right)}+l_{2}$$

When the tendon is tensioned, the finger is in the coupled-adaptive or variable coupling-adaptive grasping mode. In these two modes, when the mechanism does not contact objects, it is mainly the *OABE* of the mechanism (including linkages *c*1-2-3-5) that takes effect. The proximal phalanx and distal phalanx are a coupled relationship, and the length of tendon *BE* determines the coupling ratio. Therefore, there are dual inputs (input lever $\theta_{1}$ and tendon *BE’s* length $l_{5}$) and dual outputs (angle of the proximal phalanx $\theta_{2}$ and distal phalanx $\theta_{23}$) in this mechanism. Then, according to the geometric structure, the vector method can be used to deduce the input–output relationship as follows:

(3) $$\theta_{2}=\frac{\theta_{1}-arc\;\cos\frac{l_{1}^{2}+l_{2}^{2}-l_{3}^{2}}{2l_{1}l_{2}}}{2}$$

(4) $$\theta_{23}=\;arc\sin\frac{l_{5}^{2}-l_{c1}^{2}-l_{2}^{2}-l_{3}^{2}}{2l_{2}l_{3}}-\theta_{2}$$

When tendon BE is relaxed, or the finger mechanism contacts the object, the finger mechanism changes into the shape-adaptive mode. There is no relative motion between the proximal phalanx and distal phalanx in this mode, and the motion relationship is simple. Combining Equations (1) and (2) and then differentiating with respect to time yields the angular velocity relationship between the drive rod, the proximal phalanx, and the distal phalanx:

(5) $$\begin{array}{l} {\dot{\theta }}_{1}={\dot{\theta }}_{2}+m{\dot{\theta }}_{23}\\ m=\frac{l_{2}l_{3}\sin\theta_{23}+l_{1}l_{3}\sin\left(\theta_{1}-\theta_{2}-\theta_{23}\right)}{l_{1}l_{3}\sin\left(\theta_{1}-\theta_{2}-\theta_{23}\right)+l_{1}l_{2}\sin\left(\theta_{1}-\theta_{2}\right)} \end{array}$$

${\dot{\theta }}_{1}$ is the drive rod’s angular velocity, which is determined by the motor input speed. ${\dot{\theta }}_{2}$ and ${\dot{\theta }}_{23}$ are the angular velocity of the proximal phalanx and distal phalanx. Before the finger contacts the object, the ${\dot{\theta }}_{23}$ is 0. After the finger contacts the object, the ${\dot{\theta }}_{2}$ is 0, and tendon 5 frees up a degree of freedom to make $\theta_{23}$ move further.

### 3.2. Statics Analysis

During the analysis, the finger is considered to be an ideal constraint mechanism. The friction between the finger’s joints and the friction between the finger and the object are ignored. Compared with the linkage mechanism, tendon 5 plays a weak role in statics analysis, so the influence of the tendon is not taken into account here. The external forces acted on the finger are shown in Figure 3, including the driving moment *τ* acted on rod 1 and the contact forces $f_{1}$ and $f_{2}$ that exist between the finger and the object.

According to the principle of virtual work:

(6) $$T^{T}\omega =F^{T}v$$

In the equation:

(7) $$\begin{array}{l} T=[\tau ]\;\;,\;\;\omega =\left[\begin{array}{c} {\dot{\theta }}_{1} \end{array}\right]=\left[\begin{array}{cc} 1 & m \end{array}\right]\left[\begin{array}{c} {\dot{\theta }}_{2}\\ {\dot{\theta }}_{3} \end{array}\right]=J_{T}\dot{\theta },\;\alpha =\psi_{2}-\theta_{23}\\ F=\left[\begin{array}{c} f_{1}\\ f_{2} \end{array}\right]\;\;,\;\;v=\left[\begin{array}{c} v_{1}\\ v_{2} \end{array}\right]=\left[\begin{array}{cc} d_{1} & 0\\ l_{2}\cos\alpha +d_{2} & d_{2} \end{array}\right]\left[\begin{array}{c} {\dot{\theta }}_{2}\\ {\dot{\theta }}_{3} \end{array}\right]=J_{F}\dot{\theta } \end{array}$$

The ${\dot{\theta }}_{1}$ is the virtual angular velocity of the driving rod 1. The $v_{1}$ and $v_{2}$ are the normal virtual velocity at the contact point of the proximal phalanx and distal phalanx, respectively. The $d_{1}$ is the length between the proximal phalanx contact point with point O. The $d_{2}$ is the length between the distal phalanx contact point with point A.

According to the moment balance:

(8) $$F=J_{F}^{-T}J_{T}^{T}T$$

Then, the contact force is obtained as follows:

(9) $$f_{1}=\frac{\tau d_{2}-m\tau l_{2}\cos\alpha +m\tau d_{2}}{d_{1}d_{2}},\;f_{2}=\frac{\tau m}{d_{2}}$$

### 3.3. Optimization of Mechanism Parameters

The grasping force is required to be higher during operation; the proper grasping force is the key to ensuring stability and reliability when grasping objects [21,22]. Therefore, based on the kinematics and statics analysis, we take the grasping force as the optimization target and use the genetic algorithm to optimize the finger mechanism parameters [23,24,25,26]. Firstly, according to the human phalanx length and thickness, the values of $l_{2}$, $l_{6}$, and $l_{1}$ are set as 42 mm, 50 mm, and 8 mm, respectively. Secondly, we take the values of $l_{4}$, $l_{c1}$,$\;\varphi_{1}$, and $\varphi_{2}$ as the optimization parameters, and set the constraints: $40<l_{4}<45$, $3<l_{c1}<8$, $100{}^{\circ}\;<\;\varphi_{1}<180{}^{\circ}$, $100{}^{\circ}<\;\varphi_{2}<180{}^{\circ}$. Thirdly, we take the maximum resultant force of the proximal phalanx and distal phalanx as the optimization objective to ensure grasping stability. Hence, the objective function is:

(10) $$T=max\left(f_{1}+f_{2}\right)$$

We set the population size to 100, crossover probability to 0.8, and maximum evolutionary generation 200. The finger mechanism parameters are obtained, as shown in Table 1.

## 4. Multi-Mode Grasping Control Strategy

**Figure 4:**
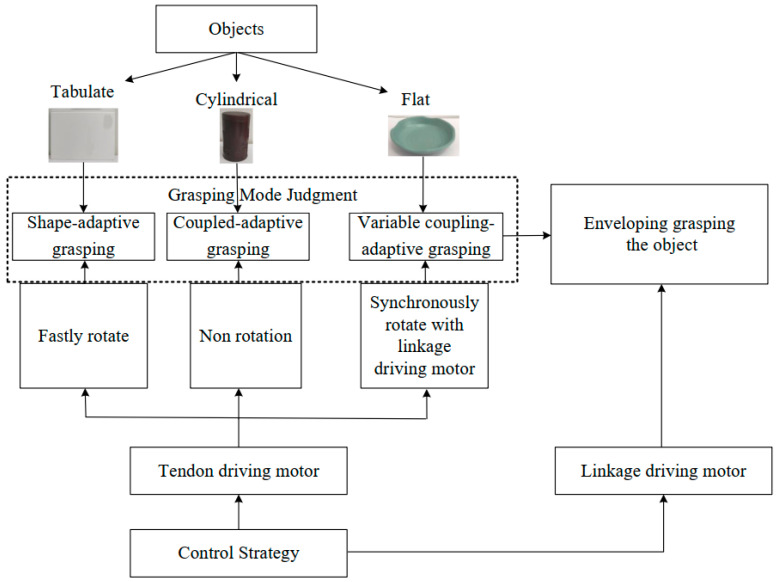
The control strategy of the finger’s three grasping modes.

The finger presented in this paper can realize three grasping modes: shape-adaptive, coupled-adaptive, and variable coupling-adaptive grasping modes. As shown in Figure 4, by controlling the speed relationship between the linkage driving motor and the tendon driving motor to adjust the tendon’s length and tension, the finger can switch between the three grasping modes. In addition, by controlling the different speed change rules of the tendon driving motor, the finger can realize different grasping trajectories.

### 4.1. Shape-Adaptive Grasping Mode

In the shape-adaptive grasping mode, there is no coupling relationship between the proximal phalanx and distal phalanx. The proximal phalanx and distal phalanx will bend as a whole before contacting objects, so it is beneficial to grasp the tabulate objects. In this mode, tendon 5 needs to remain relaxed. Therefore, before the linkage driving motor drives the finger to grasp, the tendon driving motor rotates quickly to release the tendon from the wheel, leading to the tendon becoming relaxed and losing its coupling effect.

### 4.2. Coupled-Adaptive Grasping Mode

At present, mainstream humanoid robotic hands adopt the coupled-adaptive grasping mode, in which the proximal phalanx and distal phalanx bend simultaneously with a fixed coupling ratio before contacting the object. This mode is suitable for enveloping grasping cylindrical or spherical objects. In this mode, the tendon driving motor reverse rotates to tighten the tendon, then maintains the tendon at the initial length without further rotation. The tendon is equivalent to a rigid linkage, and the entire mechanism becomes a coupled-adaptive mechanism.

### 4.3. Variable Coupling-Adaptive Grasping Mode

The finger’s distal phalanx touches the object directly and approximately vertically in the variable coupling-adaptive grasping mode, which is convenient for pinching with the thumb. Therefore, this mode is beneficial for grasping flat objects, such as bowls and plates. When the tendon motor rotates synchronously with the linkage driving motor with a specific rule, the coupling ratio between the proximal phalanx and distal phalanx dynamically changes with the tendon *BE’s* length.

**Figure 5:**
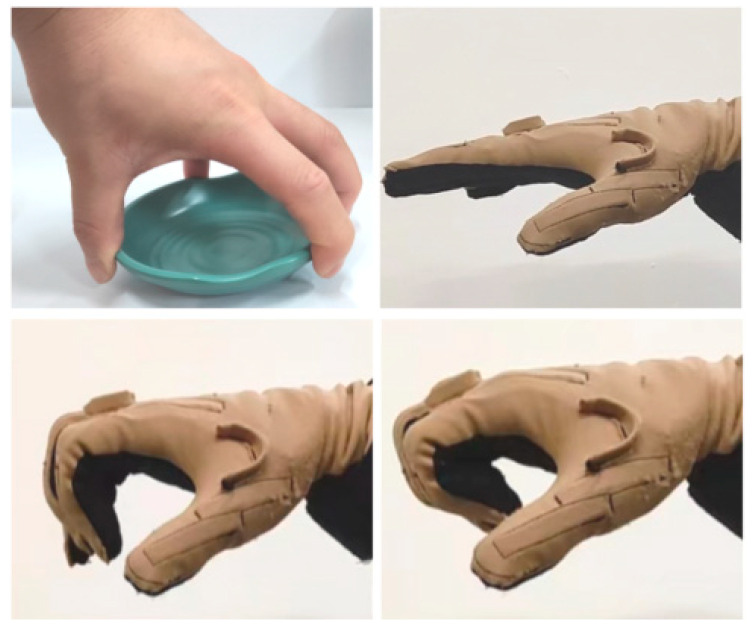
Variable coupling-adaptive grasping motion.

**Figure 6:**
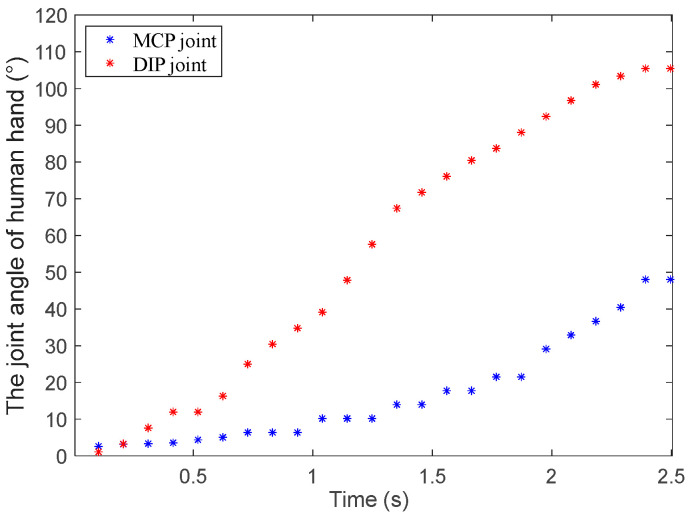
The human hand’s angle data grasping a flat saucer.

**Figure 7:**
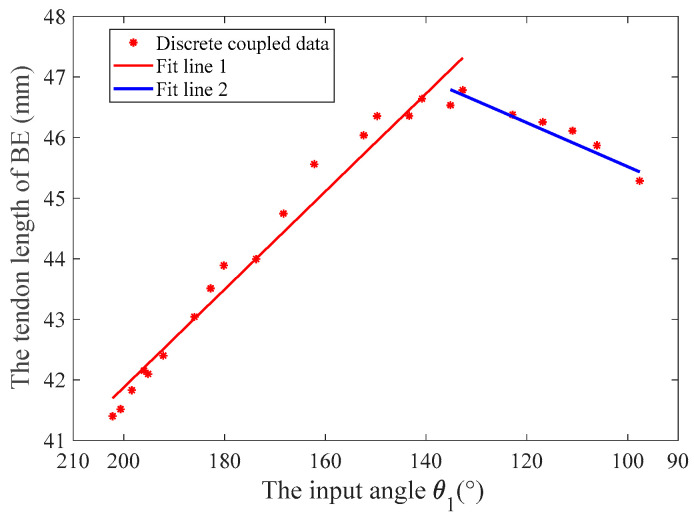
The length change rule of tendon *BE* in variable coupling-adaptive grasping mode (grasping a flat saucer, for example, as shown in Figure 5).

The variable coupling-adaptive grasping mode can achieve the motion of the input trajectory. In this mode, the length change law of tendon *BE* can be obtained through kinematic analysis and conversion to reproduce the grasping trajectory of the human hand. As shown in Figure 5, we take grasping a flat saucer as an example for analysis. Firstly, we collect the angle data of the human hand’s metacarpophalangeal (MCP) joint and proximal interphalangeal (PIP) joint from the CyberGloveII, as shown in Figure 6. Secondly, the coupling relationship between the proximal phalanx and distal phalanx is obtained from the collected data. Thirdly, we combine the result of kinematic analysis Equations (3) and (4) in Section 3. Finally, the relationship between the driving rod’s input angle $\theta_{1}$ and tendon 5’s length can be derived, as shown in Figure 7. The obtained discrete data are fitted with a polynomial curve, and a linear fitting of the first order is used in this example:

(11) $$\{\begin{array}{c} l_{BE1}=-0.081\;\theta_{1}+58.051,\;135{}^{\circ}\leq \theta_{1}<210{}^{\circ}\\ l_{BE2}=0.0361\;\theta_{1}+41.909,\;40{}^{\circ}<\theta_{1}<135{}^{\circ} \end{array}$$

Then, differentiating to time yields:

(12) $$\{\begin{array}{l} \overset{\cdot }{l_{BE1}}=-0.081\;{\overset{\cdot }{\theta }}_{1},\;135{}^{\circ}\leq \theta_{1}<210{}^{\circ}\\ \overset{\cdot }{l_{BE2}}=\;0.0361\;{\overset{\cdot }{\theta }}_{1},\;40{}^{\circ}<\theta_{1}<135{}^{\circ} \end{array}$$

We combine the drive shaft’s radius, the worm gear, and the gears’ reduction ratio to convert the speed. The speed relationship between the linkage driving motor and the tendon driving motor is obtained as follows:

(13) $$\left\{ \begin{array}{l} V_{\mathrm{tendon}}=-3.24V_{\mathrm{linkage}},\;135{}^{\circ}\leq \theta_{1}<210{}^{\circ}\\ V_{\mathrm{tendon}}=1.44V_{\mathrm{linkage}},\;40{}^{\circ}<\theta_{1}<135{}^{\circ} \end{array}\right.$$

## 5. Experimental Verification

**Figure 8:**
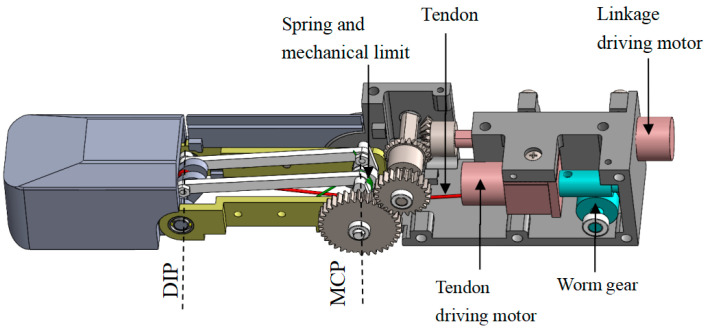
The finger’s three-dimensional internal structure.

The three-dimensional internal structure of the finger proposed in this paper is shown in Figure 8. The linkage driving motor and the tendon driving motor are placed parallel and opposite to make the structure more compact. The tendon is arranged at the symmetrical center of the finger. The proximal phalanx adopts two sets of linkage mechanisms with the center symmetrical to distribute the load evenly, reduce the risk of jamming, and increase the strength of the mechanism. A worm gear mechanism is connected at the output shaft of the tendon driving motor, which has a self-locking function to ensure the tendon cannot be stretched in the coupled-adaptive mode.

**Figure 9:**
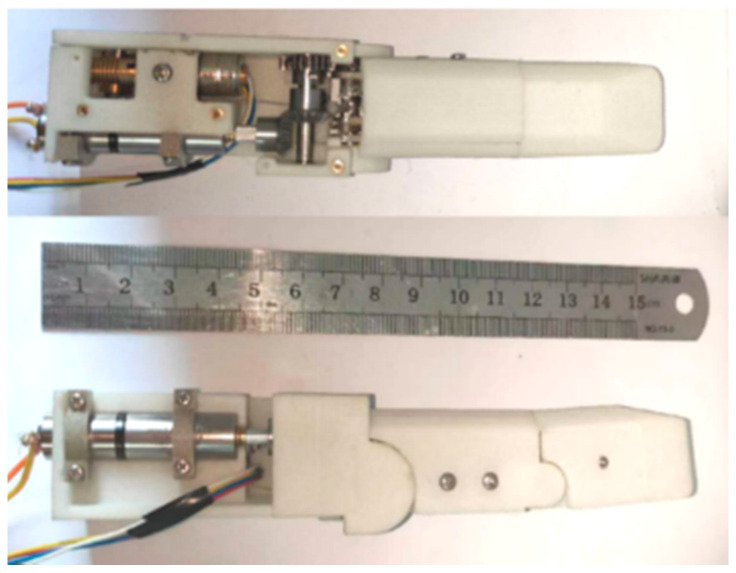
The finger’s physical model.

**Figure 10:**
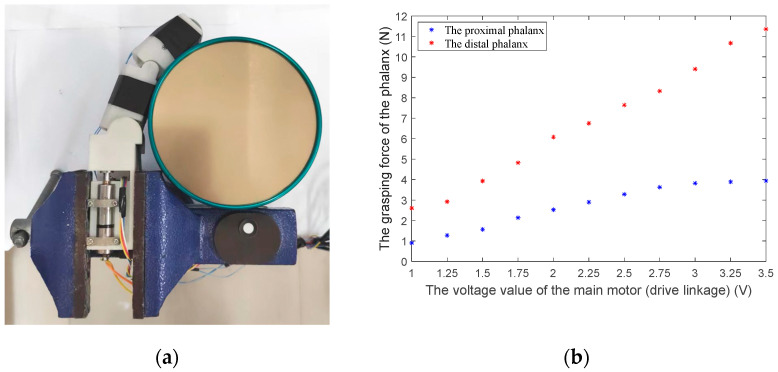
Measuring the finger’s grasping force with FSRs. (**a**) The finger arranged on a fixed platform grasps a cylindrical tin can. (**b**) The grasping force of the proximal phalanx and distal phalanx collected by the FSRs.

The prototype finger is shown in Figure 9. Considering the mechanical strength and lightweight structure requirements, the finger shell is made of glass-fiber-reinforced nylon from 3D printing, and the internal linkage mechanism is made of high-strength aluminum alloy. The finger’s overall length (including the palm part) is about 15 cm, and the phalanx length is 9 cm, which is similar to human hands. The Faulhaber micro-high-speed motor (1016K006SR) and 1024 reduction ratio reducer are used to ensure the finger‘s grasping force output. As shown in Figure 10a, we install the finger on a fixed platform. Then, we arrange two force-sensing resistors (FSRs) at the finger’s proximal phalanx and distal phalanx, respectively, to measure the finger’s grasping force when grasping a cylindrical tin can. According to Figure 10b, when the motor’s torque is approximately 0.5 N·mm with a voltage of 3.5 V, the force of the proximal phalanx and distal phalanx is approximately 4 N and 11.4 N, respectively.

**Figure 11:**
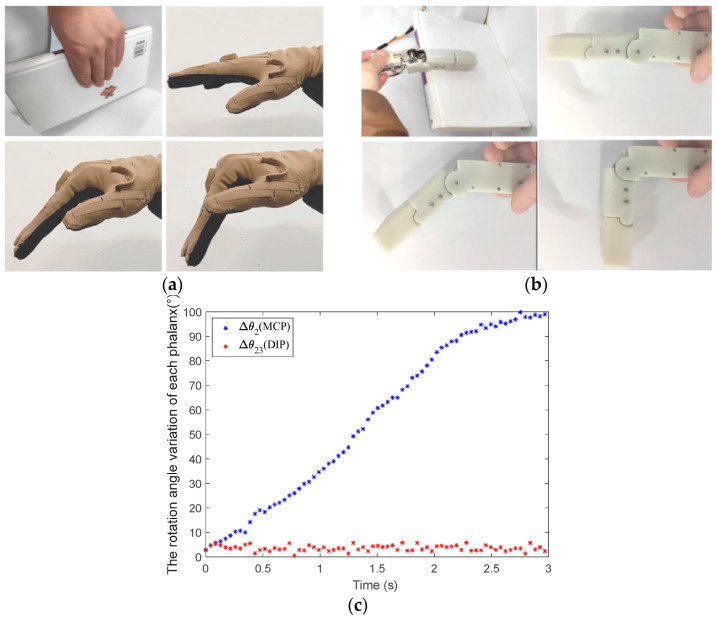
The mechanical finger adopts the shape-adaptive mode to grasp a large tabulate book. (**a**) The motion of the human hand when grasping a large tabulate book. (**b**) The motion of the mechanical finger when grasping a large tabulate book in the shape-adaptive mode. (**c**) The rotation angle of each phalanx in the shape-adaptive mode.

**Figure 12:**
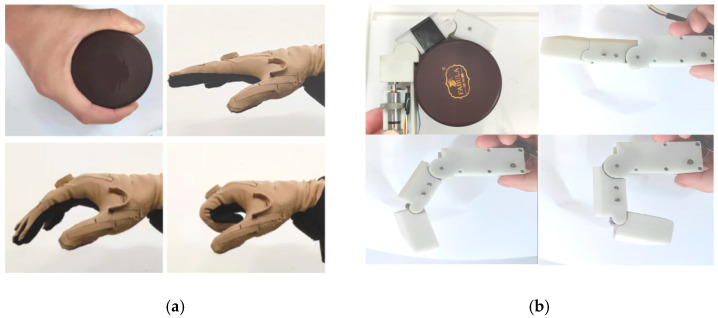

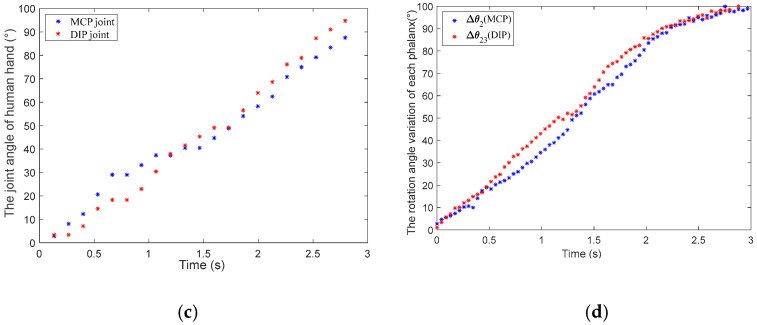
The mechanical finger adopts coupled-adaptive mode to grasp a cylindrical tin can. (**a**) The motion of the human hand when grasping a cylindrical tin can. (**b**) The motion of the mechanical finger when grasping a cylindrical tin can in the coupled-adaptive mode. (**c**) The joint angle of human hand collected by the CyberGloveII. (**d**) The rotation angle of each phalanx in the coupled-adaptive mode.

**Figure 13:**
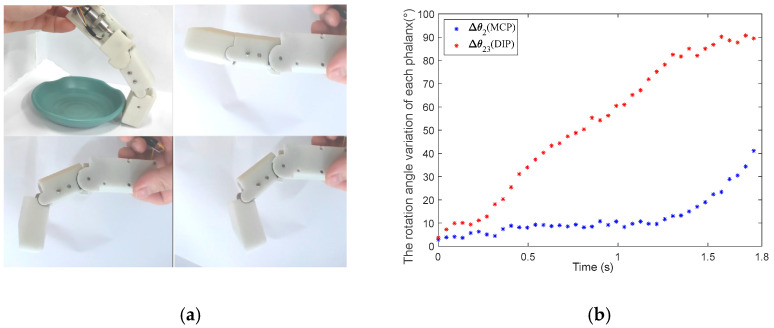
The mechanical finger adopts variable coupling-adaptive mode to grasp a flat saucer. (**a**) The motion of the human hand when grasping a flat saucer. (**b**) The rotation angle of each phalanx in the variable coupling-adaptive mode.

To verify the proposed multi-mode finger’s grasping performance, the finger’s shape-adaptive, coupled-adaptive, and variable coupling-adaptive grasping modes are tested and analyzed in this paper. We collect the finger’s MCP and distal interphalangeal (DIP) joints’ angle data using two bending sensors. Then, we compare and analyze the movement characteristics between the human hand and mechanical finger, as shown in Figure 11, Figure 12 and Figure 13.

As shown in Figure 11a, the human hand is straight when grasping a large tabulate book. There is no relative movement between the proximal phalanx and distal phalanx; they bend downward as a whole. When the tendon driving motor rotates quickly at the beginning of the finger grasp, the tendon is released from the wheel; then, the mechanical finger is in the shape-adaptive mode. The action of the finger grasping a book is shown in Figure 11b at this time. It can be seen that the grasping motion trajectory of the finger is similar to the human hand. In addition, according to the angle data collected using the bending sensor in Figure 11c, only the MCP joint of the finger rotates under the drive of the linkage driving motor, which is consistent with the human hand. Therefore, the proposed finger can grasp the large tabulate object well in shape-adaptive mode.

As shown in Figure 12a, when the human hand grasps a cylindrical tin can, it is in an enveloping grasping state. Each phalanx of the human hand is bending simultaneously and with a similar angle. If the tendon driving motor does not rotate and the tendon is initially in a tension state, the mechanical finger is in coupled-adaptive mode. Using the finger to grasp the cylindrical tin can in coupled-adaptive mode is shown in Figure 12b. It can be seen that both the finger’s MCP joint and PIP joint rotate simultaneously by comparing with Figure 12a,b. Their rotation angles are also similar according to the angle data in Figure 12d, in line with the human hand’s motion characteristics collected using CyberGloveII in Figure 12c. Therefore, the proposed finger can grasp the cylindrical or spherical object well in coupled-adaptive mode.

The human hand grasping the flat saucer is shown in Figure 5 in Section 4. The mechanical finger switches to the variable coupling-adaptive mode to grasp a flat saucer, as shown in Figure 13a. In this mode, the tendon driving motor and linkage driving motor rotate synchronously according to the speed rule obtained in Section 4. It can be seen that the distal phalanx’s grasp speed is faster than the proximal phalanx, according to Figure 13b. This motion characteristic is consistent with the human hand shown in Figure 6. Therefore, the finger has a good grasping effect under variable coupling-adaptive grasping mode.

**Figure 14:**
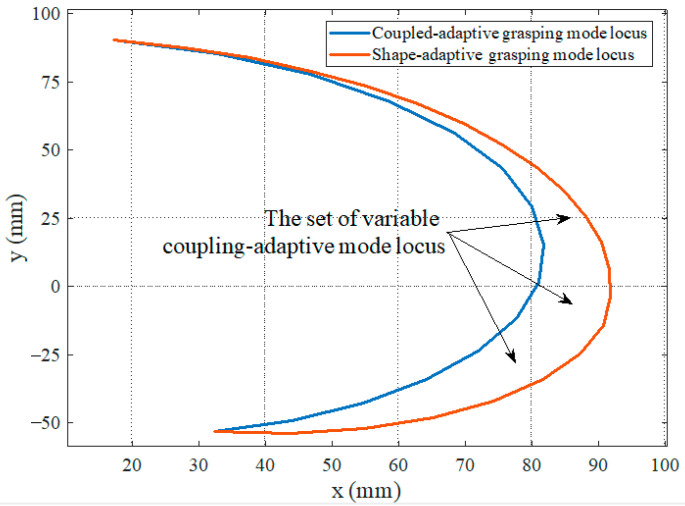
Fingertip motion locus of the three grasping modes.

**Figure 15:**
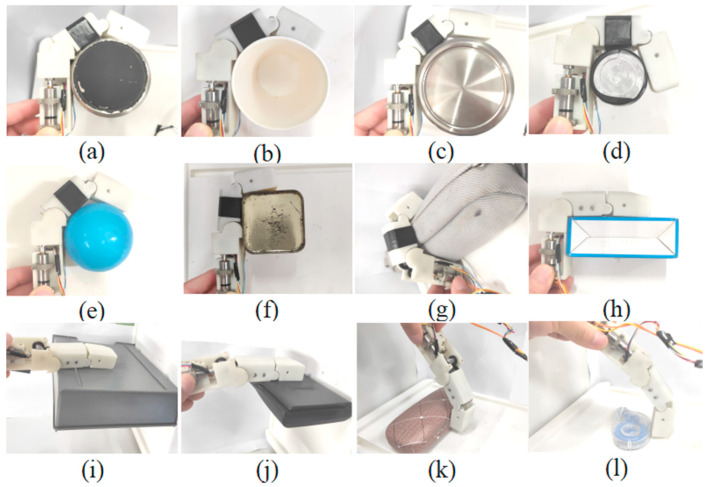
Grasping evaluation experiments. (**a**–**g**) Grasping tasks carried in the coupled-adaptive mode. (**h**–**j**) Grasping tasks carried in the shape-adaptive mode. (**k**,**l**) Grasping tasks carried in the variable coupling-adaptive mode.

Based on the above grasping experiments, the finger presented in this paper can achieve the three grasping modes. The fingertip locus of the finger in the shape-adaptive and coupled-adaptive modes is drawn through kinematics, as shown in Figure 14. It can be seen that the grasping space of the shape-adaptive grasping mode is the largest, and the coupled-adaptive grasping mode is the smallest. In addition, the grasping space of the variable coupling-adaptive grasping mode is in the middle part between them. By controlling different speed change rules of the tendon driving motor, various variable coupling-adaptive grasping modes can be realized. The finger’s grasping space and motion trajectory can be dynamically adjusted to adapt to different grasping scenes and targets, which improves the finger’s dexterity and grasping mode diversity. The grasping evaluation experiments show that the prototype finger performed well in its shape-adaptive capability and can effectively envelop various cylinders, spheres, rectangular objects, flat plates, and small parts of different diameters, as shown in Figure 15.

## 6. Conclusions

This paper presented a novel design for a multi-mode mechanical finger based on linkage and tendon fusion transmission. The finger can realize shape-adaptive, coupled-adaptive, and variable coupling-adaptive grasping. Firstly, the mechanical structure of the proposed finger was introduced. The finger’s structural parameters were optimized. Furthermore, we discussed controlling the tendon’s length and tension to switch three different modes. Subsequently, the finger’s grasping performance was verified through grasping experiments using a prototype finger.

The research demonstrates that the finger can achieve three grasping modes by controlling the relationship between the linkage driving motor and the tendon driving motor. In addition, the finger’s grasping space and motion trajectory can be dynamically adjusted by the drive speed of the tendon driving motor, which can adapt to different objects. Hence, the proposed fingers can improve the flexibility of the robotic hand and achieve grasping operations similar to humans.

In the future, we will design a multi-finger robotic hand to perform more grasping tasks. Furthermore, we will combine with the vision to identify the grasping object’s contour, then design the corresponding grasping control strategy to realize efficient multi-mode grasping operation.

## Figures and Tables

**Table 1:** The finger mechanism parameters.

Parameter	$l_{1}$	$l_{2}$	$l_{3}$	$l_{4}$	$l_{6}$	$l_{c1}^{}$	$\varphi_{1}$	$\varphi_{2}$
Value (mm)	8	42	5	41.88	50	5	150°	120°

## Data Availability

Not applicable.
